# The effect of silver impregnation of surgical scrub suits on surface bacterial contamination

**DOI:** 10.1016/j.tvjl.2011.06.039

**Published:** 2012-06

**Authors:** A.I. Freeman, L.J. Halladay, P. Cripps

**Affiliations:** School of Veterinary Science, University of Liverpool, Leahurst, Chester High Road, Neston, Cheshire CH64 7TE, UK

**Keywords:** Silver, Scrub suit, Infection control, Bacteria, Surgery

## Abstract

Silver-impregnated fabrics are widely used for their antibacterial and antifungal effects, including for clinical clothing such as surgical scrub suits (scrubs). This study investigated whether silver impregnation reduces surface bacterial contamination of surgical scrubs during use in a veterinary hospital. Using agar contact plates, abdominal and lumbar areas of silver-impregnated nylon or polyester/cotton scrubs were sampled for surface bacterial contamination before (0 h) and after 4 and 8 h of use. The number of bacterial colonies on each contact plate was counted after 24 and 48 h incubation at 37 °C. Standard basic descriptive statistics and mixed-effects linear regression were used to investigate the association of possible predictors of the level of bacterial contamination of the scrubs with surface bacterial counts.

Silver-impregnated scrubs had significantly lowered bacterial colony counts (BCC) at 0 h compared with polyester/cotton scrubs. However, after 4 and 8 h of wear, silver impregnation had no effect on BCC. Scrub tops with higher BCC at 0 h had significantly higher BCC at 4 and 8 h, suggesting that contamination present at 0 h persisted during wear. Sampling from the lumbar area was associated with lower BCC at all three time points. Other factors (contamination of the scrub top with a medication/drug, restraint of patients, working in the anaesthesia recovery area) also affected BCC at some time points. Silver impregnation appeared to be ineffective in reducing bacterial contamination of scrubs during use in a veterinary hospital.

## Introduction

Progressive contamination of clinical clothing with a mixture of bacteria from the wearer and the environment is a common occurrence (Babb et al., 1983; Boyce et al., 1997; Callaghan, 1998; Hambraeus, 1973; Loh et al., 2000; Perry et al., 2001; Speers et al., 1969; Wong et al., 1991) and is not necessarily prevented by wearing further protective clothing when contamination is perceived to be a risk (Kaplan et al., 2003). Furthermore, bacteria such as *Enterococcus* and *Staphylococcus* spp. can survive for more than 90 days on clothing worn by health care workers (Neely and Maley, 1999) and surgical scrub suits (scrubs) may be contaminated by bacteria even when freshly laundered (Moylan et al., 1975).

Although few studies have investigated the role of clinical clothing in the epidemiology of nosocomial bacterial infections, *Staphylococcus aureus* can be transferred from nurses’ uniforms to patient bedding (Hambraeus, 1973) and bacteria cultured from the front of surgical scrubs preoperatively have subsequently been isolated from infected surgical wounds (Moylan et al., 1975). Taken together, these data suggest that surgical scrubs may be a vector for the spread of bacterial infection.

Silver ions have a direct antibacterial effect (Bragg and Rainnie, 1974; Feng et al., 2000; Matsumura et al., 2003; Schreurs and Rosenberg, 1982; Yamanaka et al., 2005) and silver impregnation of fabrics (e.g. cotton and polyester) has potent antibacterial and antifungal effects (Dastjerdi and Montazer, 2010; Ilic et al., 2009b; Lee and Jeong, 2004; Matyjas-Zgondek et al., 2008; Rai et al., 2009) that persist through dying of the fabric (Ilic et al., 2009a). These properties have led to silver’s wide clinical use as an antimicrobial (Rai et al., 2009). However, some authors have questioned the utility of silver coatings in a clinical setting, since some bacterial strains may be silver-resistant (Hendry and Stewart, 1979; McHugh et al., 1975; Neely and Maley, 2000) and the coating’s effect may be altered by the presence or absence of water (Takai et al., 2002). To the authors’ knowledge there are no previously-published veterinary studies assessing the use of silver-impregnated surgical scrubs. The present study investigated whether silver-impregnated surgical scrub tops carry less surface bacteria than traditional polyester/cotton tops while being worn by veterinary staff.

## Materials and methods

Surgical nursing and anaesthesia staff in the Small Animal Teaching Hospital (SATH), University of Liverpool, were randomly assigned to wear either silver-impregnated nylon scrubs (Buckley Lamb) or standard polyester/cotton scrubs (Urban Scrubs Ltd and Lundau Scrubs) on each day of the study. These staff groups were chosen because they were typically working inside the theatre suite for the whole day, allowing the mid-abdominal and mid-lumbar surface of each scrub top to be sampled for surface bacterial contamination prior to use (0 h), after 4 h use and after 8 h use.

Four centimetre diameter agar contact plates (Irradiated tryptone soya with Tween 80, lechithin, histidine and sodium thiosulfate contact plates, Southern Group Laboratory) were placed directly onto the fabric for 1–3 s. A different, adjacent section of fabric was sampled at each of the three time points to avoid sampling areas contaminated with agar from previous plates. Before and after each sampling, the researcher’s hands were cleaned with chlorhexidine surgical scrub (Hibiscrub, AstraZeneca).

To allow statistical analysis of other possible influences on the level of bacterial contamination, participants each day completed a short questionnaire about factors that might affect how contaminated their scrubs had become (Table 1). If a participant had to change their scrub top during the course of the day (e.g. due to soiling), data for that participant for that day were excluded from the study.

Contact plates were incubated at 37 °C and the number of bacterial colonies on each plate was counted after 24 and 48 h incubation, allowing counting of both faster-growing and slower-growing colonies. Each day, two randomly-selected unused contact plates were incubated alongside the sample plates as negative controls. Both types of scrubs were laundered daily in a standard commercial washing machine (JLA 9277, JLA Limited) at 40 °C using a standard commercial biological washing powder (Persil biological washing powder, Lever Faberge), then tumble dried in a commercial drier (JLA 92830ELG). Both types of scrubs were folded and returned to the theatre changing area immediately after laundry and stored in open plastic baskets overnight.

To satisfy requirements for ethical approval, at the end of the study participants completed a short anonymous debriefing questionnaire about their participation. Each set of scrubs, participant, contact plate and questionnaire was assigned a unique identification number and all collected data were recorded against these numbers, ensuring that the researcher performing the bacterial colony counting was blinded to the type of scrub suit and participant’s identity and job type.

### Statistical analysis

Bacterial colony counts were recorded on a spreadsheet (Microsoft Excel) and transferred into STATA11 (StataCorp) for statistical analysis.

Data from contact plates where bacterial overgrowth had occurred and individual colonies could not be counted were coded as missing. If a contact plate contained 100 or more colonies, an accurate count could not be determined due to merging of adjacent colonies, so counts from these plates were recorded as ‘100+’ and recoded to a value of 150 before statistical analysis. All counts were transformed to log_10_(count + 1). After performing standard basic descriptive statistics, the association of the log_10_ transformed cell count with possible predictors of the level of bacterial contamination of the scrubs (questionnaire data as shown in Table 1, participant’s job role and the scrub type) was explored. Two approaches were considered: the first method offered all possible predictors to a backwards stepwise multiple regression model, retaining only those variables if their exclusion was significant at *P* ⩽ 0.2. These variables were then included in a mixed-effects linear regression model with staff identity and/or scrub suit identity as a random variable: the type of scrub suit (silver or not) was also forced into the model.

The second method included all possible predictors in a mixed-effects linear regression. In both cases, the only random effect needed by the model was staff identity. The Wald statistic was used to determine the significance of predictors in the final model. Graphical methods were used to examine the residuals for normality and equality of variance, revealing that the required assumptions were met. Since the two approaches gave very similar results, only the second is reported here. Predicted means were obtained using the STATA ‘margins’ command using its default settings. For a given level of a factor (e.g. type of scrub suit), the predicted mean provided values estimated for the model that would have been obtained if all individuals had this level of the factor. Statistical significance was defined as *P* < 0.05 on a two-sided null hypothesis.

All participants gave fully informed consent to take part in the study and were informed that they could withdraw from the study at any time for any reason. The study was approved by the University of Liverpool Research Ethics Committee.

## Results

Mixed-effects linear regression revealed that, at 0 h, samples taken from silver-impregnated scrubs had significantly lower BCC after both 24 and 48 h incubation (Tables 2–4). In samples taken at 4 and 8 h, silver impregnation had no effect on BCC (Tables 2 and 3). However, scrub tops with higher BCC at 0 h had significantly higher BCC at 4 and 8 h (Table 4). Samples taken from the lumbar area were associated with significantly lower BCC after 24 and 48 h incubation at all three sampling times (Tables 2–4). In samples taken at 4 h (after 48 h incubation) and 8 h (after 24 or 48 h incubation), contamination of the scrub top with a medication/drug was associated with significantly increased BCC (Table 4).

Restraint of patients during the day was associated with increased BCC in samples taken at 4 h and incubated for 24 or 48 h (Table 4). Two of the possible predictors were associated with lower BCC (Table 4): working in the anaesthesia recovery area during the day (in samples taken at 4 h incubated for 24 or 48 h) and contamination of the scrub top with urine (in samples taken at 8 h incubated for 48 h).

## Discussion

Although the antibacterial and antifungal properties of silver-impregnated fabrics are well known (Dastjerdi and Montazer, 2010; Ilic et al., 2009b; Lee and Jeong, 2004; Matyjas-Zgondek et al., 2008; Rai et al., 2009), our study suggests that silver impregnation is ineffective in reducing surface bacterial contamination of surgical scrub tops during use in a veterinary hospital. Data from human health care suggests that clean uniforms are progressively contaminated with a mixture of bacteria from the wearer, patients and the environment (Babb et al., 1983; Boyce et al., 1997; Callaghan, 1998; Hambraeus, 1973; Loh et al., 2000; Perry et al., 2001; Speers et al., 1969; Wong et al., 1991), with approximately one-third of bacterial contamination coming from the wearer (Speers et al., 1969). Observation of the contact plates in our study revealed a mix of colonies varying in shape (round to filamentous), colour (cream to bright yellow) and size (1–5 mm diameter), consistent with a mixed population of bacteria.

In our study, sampling from the abdominal area of the scrubs was consistently associated with higher BCC than sampling from the lumbar area and patient restraint was associated with increased BCC. This suggested that patients and the wearer’s hands were the main source of contamination of the scrub tops, since the abdominal area is most likely to come into contact with the patient during restraint or lifting and is more likely than the lumbar area to be touched by the wearer. Identification of the bacteria contaminating the scrubs might have allowed confirmation of the source of contamination but the large number of colonies grown on the contact plates precluded their identification in this study.

Silver appears to exert its antibacterial effect by several mechanisms, including denaturation and condensation of DNA (Feng et al., 2000), denaturation of ribosomes and reduced expression of respiratory enzymes (Yamanaka et al., 2005), direct interaction with sulfhydryl groups on proteins (Feng et al., 2000), generation of reactive O_2_ species and damage to the cell envelope (Feng et al., 2000). Together, these prevent DNA replication (Feng et al., 2000), inhibit bacterial respiration (Bragg and Rainnie, 1974) and cause altered metabolism of various substances, including phosphate, mannitol, succinate, glutamine and proline (Schreurs and Rosenberg, 1982).

Exposure to silver ions takes several hours to significantly reduce bacterial numbers (Yamanaka et al., 2005), suggesting that silver’s lack of efficacy in our study may have been due to ongoing contamination of the scrubs (e.g. from patient contact during restraint or movement of patients around the theatre suite) resulting in contamination with bacteria which the silver in the fabric had insufficient time to kill before sampling took place. This hypothesis is supported by the observation that silver impregnation was associated with lower BCC at 0 h, suggesting that silver was effective in reducing surface bacterial contamination during storage of the scrubs.

An alternative hypothesis is that some of the bacteria contaminating the scrubs were silver-resistant. Silver resistance has been identified in several strains of bacteria (Hendry and Stewart, 1979; McHugh et al., 1975; Neely and Maley, 2000) and is apparently conferred by a plasmid (pMG101) carrying genes for a periplasmic silver-binding protein, two transmembrane silver efflux pumps and a combination of a membrane kinase and transcriptional regulatory responder that together act as a ‘silver sensor’ (Silver, 2003). However, previous reports suggest that the levels of silver resistance in bacteria isolated from human and veterinary patients are low (Ip et al., 2006; Woods et al., 2009) and, if a substantial number of our hospital population of bacteria were silver-resistant, one might also expect BCC at 0 h to be unaffected by silver impregnation. Further work to investigate the prevalence of silver resistance in our hospital population of bacteria could clarify this issue.

Interestingly, some bacteria were present on the surface of both types of scrubs at 0 h. Although bacteria may survive laundry of scrubs (Moylan et al., 1975), evidence from human healthcare suggests that the laundry process employed here should have been effective in killing most common pathogens (Jurkovich, 2004; Patel et al., 2006). We therefore consider it likely that the majority of the bacteria detected at 0 h were acquired during handling and storage of the scrubs post-laundry or from the wearer’s hands during the process of dressing, with the relative importance of these two sources depending on the scrub type. Higher BCC at 0 h were associated with higher bacterial counts at later sampling times, suggesting that at least some of the bacteria identified in later samples were present on the scrubs when they were first put on. This indicates that surgical scrubs should be laundered, stored and donned as hygienically as possible, since bacterial contamination acquired at these times may persist while the scrubs are in use.

Contamination of the scrub top with a medication or drug was associated with increased BCC in samples taken after 4 and 8 h wear. This is surprising, since medications should be sterile and therefore not a major source of bacterial contamination. Due to the nature of the drugs being handled (e.g. anaesthetic induction agents, intravenous antibacterials) medications in our theatre suite are usually only handled for a short period around the time that they are administered to the patient. Thus, contact with medications implies close patient contact and thus possibly increased scrub suit contamination. This hypothesis is supported by the association between handling of patients and increased BCC in the samples taken after 4 h wear.

Previous data have suggested that one of the other predictors associated with patient contact (visible contamination of the scrubs with organic material such as hair/fur, vomit or faeces) should be associated with increased BCC, since contamination with organic materials reduces the effectiveness of silver as an antibacterial agent (Takai et al., 2002). In our study, this association was not identified, however, possibly because transfer of bacteria to the surface of the scrubs from the patient or environment occurred without visible contamination with these organic materials. Also, SATH uniform and work-wear guidelines state that clothing visibly contaminated with organic matter should be changed immediately, which would remove the effect of these types of contamination from the study because data from scrubs changed during the day were excluded from the analysis. Alternatively, it is possible that workers in perceived ‘high-risk’ areas, or carrying out activities with a perceived increased risk of contamination, worked more carefully or took additional precautions, such as wearing disposable plastic aprons. This might also account for the association between working in the anaesthesia recovery area (an area where patients were frequently handled while kennelled and thus perhaps with a high perceived risk of contamination) and lower BCC in samples taken after 4 h wear. However, it is also possible that some associations were missed due to limitations of the study (see below). Further work visually monitoring staff movements and patient contacts might clarify why contamination with a medication resulted in increased BCC.

Contamination of the surgical scrubs with urine was associated with lower BCC in samples taken at 8 h and incubated for 48 h. We believe this association to be due to chance and not causal because it only occurred at this one time point and visible contamination of scrubs with urine should have prompted the scrub top to be changed, thus excluding data from it on that day from the study. Also, there may have been errors in reporting (see below).

There are several possible limitations to this study. First, the number of samples in each group was lower than that assumed in the power calculation, possibly resulting in type II errors; this may account for restraint of patients being associated with higher BCC in samples taken at 4 h, but not at 8 h, for example. Unfortunately, more samples could not be taken in the time available for the study. Second, although the questionnaires were completed anonymously (to encourage full disclosure) and as soon as was practical (at the end of the working day), there may have been errors or omissions in the responses, leading to over or under-reporting of some types of contamination. However, any such errors should not alter any effects based on scrub type, sampling location, participant identification or date, since the accuracy of these data was not dependent on reporting by the participants. Third, the difficulty in counting large numbers of bacterial colonies due to colony merging and overgrowth may have resulted in underestimation of the number of colonies on some sample plates, which may have affected the results.

## Conclusions

This study suggests that silver impregnation of fabric is ineffective in reducing surface bacterial contamination of surgical scrub tops during use in a veterinary surgical suite. Furthermore, bacterial contamination of scrub suits present when they are first put on may persist through the working day, although silver impregnation does appear to be effective in reducing bacterial contamination on the scrubs during storage. The apparent lack of efficacy of silver in the face of ongoing bacterial contamination may be clinically significant, since in a busy theatre suite bacteria could be transferred between patients via the scrubs. Precautions such as wearing disposable plastic aprons during patient contact, regularly changing surgical scrubs during the working day and strict hand hygiene may be more effective in reducing bacterial contamination than buying specialist ‘antibacterial’ scrubs.

## Conflict of interest statement

None of the authors of this paper has a financial or personal relationship with other people or organisations that could inappropriately influence or bias the content of the paper.

## Figures and Tables

**Table 1:** Data recorded in daily participant questionnaire.

Information

Participant identification
Scrub suit identification
Date
Length of time working in theatre suite (to nearest half hour)
If participant had restrained patients
If participant had prepared animals for surgery (clipping and scrubbing)
If participant left the theatre suite to go to the imaging suite while wearing scrubs and, if so, how many times and for how long (to the nearest half hour)
Whether or not the front of the participant’s scrub top had been contaminated with blood, surgical scrub solution, urine, faeces, vomit, saliva, hair/fur, food, water or medications/drugs
If the participant was present in one of the operating theatres or procedure rooms during surgery and, if so, whether they were an observer, theatre nurse, anaesthetist or scrubbed surgical assistant.
Which areas of the theatre suite the participant had worked in (theatres 1–3, minor procedures theatre, endoscopy room, anaesthesia induction area, anaesthesia recovery area, intensive care unit and cleaning/sterilisation area)
Whether the participant had to change their scrub top during the day

**Table 2:** Uncorrected raw means, medians, standard errors of the mean (SEM) and interquartile ranges (IQR) of log_10_ (bacterial colony count + 1) for normal and silver-impregnated scrubs and abdominal and lumbar sampling locations at sampling times of 0, 4 and 8 h. Number of observations ranged from *n* = 115 to *n* = 127.

Sampling time (h)	Sample source	Incubation time (h)	Mean	SEM	Median	IQR
0	Normal scrubs	24	1.02	0.045	0.95	0.58
		48	1.10	0.044	1.04	0.60
	Silver scrubs	24	0.85	0.053	0.85	0.73
		48	0.95	0.050	0.87	0.68
	Abdominal area	24	1.15	0.047	1.11	0.75
		48	1.23	0.044	1.23	0.68
	Lumbar area	24	0.72	0.044	0.70	0.52
		48	0.81	0.042	0.78	0.56

4	Normal scrubs	24	1.20	0.047	1.18	0.67
		48	1.27	0.046	1.23	0.70
	Silver scrubs	24	1.27	0.045	1.28	0.70
		48	1.34	0.042	1.34	0.61
	Abdominal area	24	1.45	0.040	1.38	0.54
		48	1.52	0.038	1.52	0.53
	Lumbar area	24	1.02	0.043	1.02	0.62
		48	1.11	0.041	1.08	0.54

8	Normal scrubs	24	1.25	0.046	1.22	0.60
		48	1.31	0.043	1.26	0.57
	Silver scrubs	24	1.17	0.041	1.15	0.69
		48	1.23	0.042	1.18	0.72
	Abdominal area	24	1.40	0.042	1.40	0.63
		48	1.46	0.040	1.46	0.58
	Lumbar area	24	1.02	0.038	0.98	0.45
		48	1.09	0.038	1.04	0.43

**Table 3:** Predicted means and 95% confidence intervals for normal and silver-impregnated scrubs and abdominal and lumbar sampling locations at sampling times of 0, 4 and 8 h. estimated after mixed-effects linear regression analysis of log_10_ (bacterial colony count + 1). Number of observations ranged from *n* = 115 to *n* = 127.

Sampling time (h)	Sample source	Incubation time (h)	Predicted mean	95% Confidence interval
0	Normal scrubs	24	1.071	0.910–1.233
		48	1.142	0.988–1.296
	Silver scrubs	24	0.896	0.738–1.053
		48	0.983	0.833–1.133
	Abdominal area	24	1.197	1.039–1.355
		48	1.276	1.125–1.427
	Lumbar area	24	0.771	0.613–0.929
		48	0.85	0.700–1.001

4	Normal scrubs	24	1.181	1.041–1.320
		48	1.246	1.138–1.355
	Silver scrubs	24	1.243	1.107–1.379
		48	1.352	1.247–1.457
	Abdominal area	24	1.391	1.254–1.528
		48	1.479	1.375–1.583
	Lumbar area	24	1.036	0.901–1.171
		48	1.128	1.026–1.231

8	Normal scrubs	24	1.253	1.137–1.369
		48	1.321	1.212–1.430
	Silver scrubs	24	1.149	1.035–1.264
		48	1.209	1.102–1.316
	Abdominal area	24	1.343	1.229–1.457
		48	1.412	1.306–1.518
	Lumbar area	24	1.068	1.229–1.457
		48	1.125	1.022–1.228

**Table 4:** Statistically significant results from mixed-effects linear regression analysis of effect of possible predictors on log_10_ (bacterial colony count + 1) at sampling times of 0, 4 and 8 h. A negative coefficient indicates that the presence of the predictor reduces bacterial colony count, whereas a positive coefficient indicates that presence of the predictor increases colony count. Number of observations ranged from *n* = 115 to *n* = 127.

Sampling time (h)	Possible predictor	Incubation time (h)	Coefficient	*P* value	95% Confidence interval
0	Silver impregnation of scrubs	24	−0.176	0.004	−0.296 to −0.055
		48	−0.159	0.006	−0.271 to −0.046
	Sample taken from lumbar area of scrub top	24	−0.426	0.000	−0.538 to −0.314
		48	−0.425	0.000	−0.530 to −0.320

4	Colony count at time 0	24	0.155	0.014	0.032–0.278
		48	0.132	0.037	0.008–0.257
	Sample taken from lumbar area of scrub top	24	−0.359	0.000	−0.475 to −0.244
		48	−0.352	0.000	−0.465 to −0.240
	Wearer restrained patients	24	0.360	0.007	0.099–0.620
		48	0.327	0.011	0.074–0.578
	Wearer entered anaesthesia recovery area	24	−0.235	0.029	−0.446 to −0.024
		48	−0.266	0.010	−0.469 to −0.063
	Scrub top contaminated with a medication	48	0.211	0.021	0.032–0.391

8	Colony count at time 0	24	0.207	0.001	0.087–0.327
		48	0.177	0.005	0.054–0.300
	Sample from lumbar area of scrub top	24	−0.281	0.000	−0.394 to −0.168
		48	−0.289	0.000	−0.400 to −0.177
	Scrub top contaminated with a medication	24	0.216	0.018	0.037–0.395
		48	0.234	0.009	0.057–0.410
	Scrub top contaminated with urine	48	−0.187	0.032	−0.357 to −0.016
